# Update on the management of acute pharyngitis in children

**DOI:** 10.1186/1824-7288-37-10

**Published:** 2011-01-31

**Authors:** Marta Regoli, Elena Chiappini, Francesca Bonsignori, Luisa Galli, Maurizio de Martino

**Affiliations:** 1 Department of Sciences for Woman and Child's Health, University of Florence, Florence, Italy

## Abstract

Streptococcal pharyngitis is a very common pathology in paediatric age all over the world. Nevertheless there isn't a joint agreement on the management of this condition. Some authors recommend to perform a microbiological investigation in suspected bacterial cases in order to treat the confirmed cases with antibiotics so to prevent suppurative complications and acute rheumatic fever. Differently, other authors consider pharyngitis, even streptococcal one, a benign, self-limiting disease. Consequently they wouldn't routinely perform microbiological tests and, pointing to a judicious use of antibiotics, they would reserve antimicrobial treatment to well-selected cases. It has been calculated that the number of patients needed to treat to prevent one complication after upper respiratory tract infections (including sore throat), was over 4000.

Even the use of the Centor score, in order to evaluate the risk of streptococcal infection, is under debate and the interpretation of the test results may vary considerably. Penicillin is considered all over the world as first line treatment, but oral amoxicillin is also accepted and, due to its better palatability, can be a suitable option. Macrolides should be reserved to the rare cases of proved allergy to β-lactams. Cephalosporins can be used in patients allergic to penicillin (with the exception of type I hypersensibility) and have been also proposed to treat the relapses.

## Introduction

Acute pharyngitis is defined as an infection of the pharynx and/or tonsils. It is a very common pathology among children and adolescents. Although viruses cause most acute pharyngitis episodes, group A Streptococcus (GABHS) causes 37% of cases of acute pharyngitis in children older than 5 years [1]. Other bacterial causes of pharyngitis are Group C *Streptococcus *(5% of total cases), *C. pneumoniae *(1%), *M. pneumoniae *(1%) and anaerobic species (1%). Between viruses Rhinovirus, Coronavirus and Adenovirus account for the 30% of the total cases, Epstein Barr virus for 1%, Influenza and Parainfluenza virus for about 4% [2].

Streptococcal pharyngitis has a peak incidence in the early school years and it is uncommon before 3 years of age. Illness occurs most often in winter and spring [3]. The infection is transmitted via respiratory secretions and the incubation period is 2-5 days. Communicability of the infection is highest during acute phase and in untreated people gradually diminishes over a period of weeks; it ceases after 24 hours of antibiotic therapy [4].

Clinical manifestations include sore throat and fever with sudden onset, red pharynx, enlarged tonsils covered with a yellow, blood-tinged exudate. There may be petechiae on the soft palate and posterior pharynx. The anterior cervical nodes are enlarged and swollen. Headache and gastrointestinal symptoms (vomiting and abdominal pain) are frequent. Table 1 shows signs and symptoms of GABHS pharyngitis and their sensitivity and specificity for the diagnosis [5].

**Table 1:** Clinical signs and symptoms of GABSH pharingitis , their sensitivity and specificity [5]

Symptoms and Clinical Findings	Sensitivity (%)	Specificity (%)
**Absence of cough**	51-79	36-68

**Anterior cervical nodes swollen or enlarged**	55-82	34-73

**Headache**	48	50-80

**Myalgia**	49	60

**Palatine petechiae**	7	95

**Pharyngeal exudates**	26	88

**Fever >38°C**	22-58	52-92

**Tonsillar exudate**	36	85

The onset of viral pharyngitis may be more gradual and symptoms more often include rhinorrhea, cough, diarrhea, hoarseness. Several clinical scores have been proposed to help the clinician in the diagnosis; they are illustrated in table 2.

**Table 2:** Clinical Score for GABSH pharyngitis.

Reference	Clinical signs and symptoms	Sensibility (%)	Specificity (%)
[37]	Recent exposure to GABHS, pharyngeal exudate, enlarged or tender cervical nodes, fever	55	74

[38]	Season, age, white cells count, fever, absence of cough, enlarged cervical nodes, tonsillar exudate or swelling	68	85

[39]	Swollen and tender anterior cervical nodes, tonsillar exudate	84	40

[40]	Fever, cervical nodes enlargement, tonsillar exudate or swelling or hypertrophy, Absence of cough	63	67

[41]	Season, age, fever, enlarged cervical nodes, tonsillar exudate or swelling or hypertrophy, absence of cough or rhinitis or conjunctivitis	22	93

[42]	Tonsillar hypertrophy, enlarged cervical nodes, absence of rhinitis, scarlet fever rash	18	97

Anyway the clinical presentations of GABHS and viral pharyngitis show considerable overlap and no single element of the patient's history or physical examination reliably confirms or excludes GABHS pharyngitis [5].

Complications of the infection can be distinguished in suppurative and nonsuppurative. Suppurative complications, due to the spread of GABHS to adjacent tissues, include cervical lymphadenitis, peritonsillar abscess, retropharyngeal abscess, otitis media, mastoiditis and sinusitis. The use of antibiotics have reduced the incidence of this group of complications, that remain a reality when primary illness has gone unnoticed or untreated [3].

Not suppurative, immune-mediated *sequelae *are acute rheumatic fever (ARF), acute post-streptococcal glomerulonephritis, Sydenham chorea, reactive arthritis and Paediatric Autoimmune Neuropsychiatric Disorders Associated with *Streptococcus pyogenes*.

According to WHO, at least 15.6 million people have rheumatic hearth disease (RHD), and 233 000 deaths annually are directly attributable to ARF. Due to the limitations of reports related to limited resources in developing countries, it is likely that the prevalence and incidence of ARF are largely underestimated [6].

The prevalence of RHD in children aged 5-14 years is higher in sub-Saharan Africa (5.7 per 1000), in Indigenous populations of Australia and New Zealand (3.5 per 1000), and southcentral Asia (2.2 per 1000), and lower in developed countries (usually 0.5 per 1000) [7].

A systematic review of 10 population-based studies from 10 countries on all continents, except Africa, published from 1967 to 1996, describes the worldwide incidence of ARF. The overall mean incidence rate of first attack of ARF was 5-51/100,000 population (mean 19/100,000; 95% CI 9 to 30/100,000). A low incidence rate of ≤10/100,000 per year was found in America and Western Europe, while a higher incidence (> 10/100,000) was documented in Eastern Europe, Middle East (highest), Asia and Australasia. Studies with longitudinal data displayed a falling incidence rate over time [8].

In the United States, the number of ARF cases has fallen dramatically over the last half century. A national study conducted in 2000 detailing the characteristics of American pediatric patients hospitalized with ARF found that the incidence was 14.8 cases per 100,000 hospitalized children (though the true national incidence of ARF cases is 1 case per 100,000 population) [9].

The diagnosis of GABHS pharyngitis can be done by a throat culture or rapid diagnostic test for GABHS (RADT). The culture is the gold standard for diagnosis but requires 18-24 hours of incubation at 37°C, causing a delay in identification of GABHS. This delay in diagnosis often leads physicians to administer therapy without first knowing the etiological agent, causing an overuse of antibiotics that provokes a rising in the diffusion of drug-resistant bacterial strains. RADTs allow the identification of GABHS on a throat swab in a matter of minutes. This strategy has a significant impact on reducing the antibiotic prescription [10]. The tests are based on nitrous acid extraction of group A carbohydrate antigen from organisms obtained by throat swab. The specificities of RADTs are generally high while sensitivities vary considerably [4]. Rapid tests offer good accuracy for use as diagnostic method, however, in some situations, they have to be complemented with the microbiological culture, because of the possibility of false negative results [11]. Tanz *et al *in a study including 1848 children from 3 to 18 years evaluated for acute pharyngitis in 6 community pediatric offices demonstrate that Rapid antigen-detection test sensitivity was 70%. Office culture sensitivity was significantly greater, 81%. Rapid antigen-detection test specificity was 98%, and office culture specificity was 97%, a difference that was not statistically significant [12].

## Management

There is no joint agreement for the clinical management of pharyngotonsillitis. Experts recommendations and guidelines differ considerably regarding how to make diagnosis, weather and when to treat. The many opinions can be summarized in two position. One position, espoused by American [4,13-15], French [16] and Finnish [17] experts, considers GABHS pharyngitis an infection that need to be recognised and treated to avoid complications, first of all ARF. This implies the recommendation to perform microbiological tests for detecting the bacterial forms in order to treat them. According to this position, in Italy a regional guideline has been developed in Emilia Romagna [18]. The other position, followed by UK [19], Scottish [20], Dutch [21] and Belgian [22] authors, considers pharyngitis, even GABHS one, a benign self limiting disease, given to the low incidence of suppurative complications and ARF in developed countries. This second idea leads to reserve antibiotics treatment to selected cases, so as to make a judicious use of antibiotics in order to avoid the spread of resistant strains.

According to this position, a big retrospective cohort study conduced by Petersen *et al*. in UK primary care practices, on a total number of 3.36 million episodes of respiratory tract infection, found out that the number of patients needed to treat to prevent one complication after upper respiratory tract infections (including sore throat and otitis media), was over 4000. The study concludes that antibiotics are not justified to reduce the risk of serious complications for upper respiratory tract infection, sore throat, or otitis media [23].

We are going to examine the different perspectives on the management of pharyngitis in order to analyze substantial differences.

## Diagnosis and indications to treat

With regard to diagnosis, major disputes concern the use of microbiological tests (throat culture or RADT). A clinical score proposed by Centor and subsequently modified, considers the combination of signs and symptoms suggestive of GABHS pharyngitis and could help clinician to address diagnosis [24]. Table 3 shows the Centor score.

**Table 3:** Centor Score [24].

Clinical criteria	Points
Absence of cough	1

Swollen and tender anterior cervical nodes	1

Temperature > 38°C	1

Tonsillar exudate or swelling	1

Age 3 to 14 years	1

Age 15 to 44 years	0

Age 45 years and older	-1

Anyway clinical of GABHS and viral pharyngitis can be overlapped and no single element of the patient's history or physical examination reliably confirms or excludes GABHS pharyngitis [5]. The indication to make diagnosis using the Centor score alone or in association with microbiological tests varies widely all over the world.

**Figure 1:**
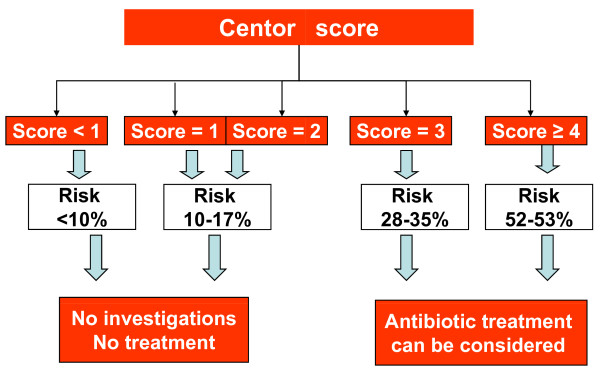
**NICE guideline: flow-chart for management of pharyngitis **[19].

English experts in NICE guidelines state that, depending on clinical assessment of severity, patients presenting acute pharyngitis can be considered for an immediate antibiotic prescribing strategy (in addition to a no antibiotic or a delayed antibiotic prescribing strategy) if three or more Centor criteria are present. Otherwise if Centor ≤ 2, no further investigations and no treatment are required [19]. UK guidelines reserve an immediate antibiotic prescription or further investigations to the situations in which the patient is systemically very unwell, has symptoms and signs suggestive of serious illness or suppurative complications, or when a pre-existing comorbidity (significant heart, lung, renal, liver or neuromuscular disease, immunosuppression, cystic fibrosis, and young children who were born prematurely) is present [19]. Figure 1 shows the flow chart of UK guideline (NICE guideline) [19]. Equally Scottish authors suggest that neither throat swabs nor rapid antigen testing should be carried out routinely in sore throat even if clinical examination is not considered reliable upon to differentiate between viral and bacterial etiology. Scottish experts consider that the prevention of suppurative complications and ARF is not a specific indication for antibiotic therapy in pharyngitis [20].

On the other hand, most of the American authors suggest the necessity of a microbiological confirmation for the diagnosis of GABHS; clinical criteria can help clinician to select patients who need to be tested [4,13-15].

**Figure 2:**
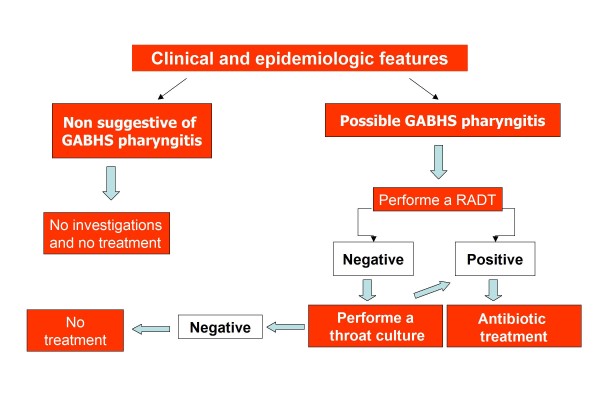
**IDSA guideline: flow-chart for management of pharyngitis **[14].

**Figure 3:**
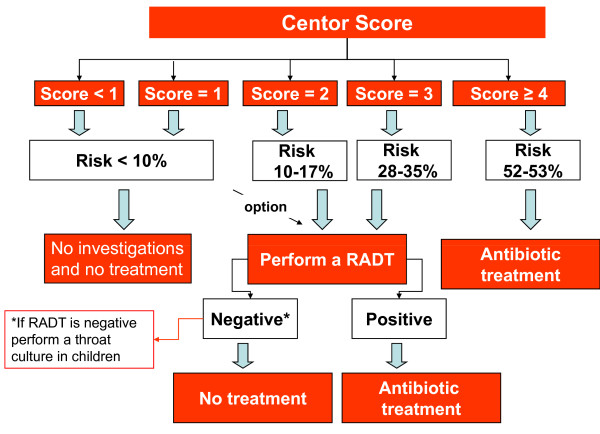
**ACP guideline: flow-chart for management of pharyngitis **[15].

Bisno *et al*, in the Infectious Diseases Society of America (IDSA) guidelines, state to identify patients who may have GABHS pharyngitis considering clinical and epidemiological features. If clinical and epidemiological features suggest the possibility of GABHS infection, a laboratory test (culture or RADT) should be performed and, in case of positivity, antibacterial treatment should be prescribed to the patient [14]. Figure 2 shows the flow chart recommended by Bisno *et al *in IDSA guidelines [14]. Snow *et al*, in the American College of Physicians (ACP) guidelines, suggest the use of Centor score to identify patients who may have GABHS pharyngitis. If Centor score is ≥ 2, then a microbiological test should be performed. Adult patients with a Centor score ≥ 4 should be treated without needing microbiological confirmation [15]. The flow chart suggested by Snow *et al *in ACP guidelines is illustrated in figure 3[15]. However, it has been argued that this latter approach would result in an over-treatment since only 50% of patients with a Centor score of 4 suffer from a streptococcal pharyngitis [25].

Gerber *et al *in the scientific statement from the American Heart Association, suggest to screen patients with clinical and epidemiological criteria and to perform RADT or throat culture in all patients with risk [13].

Focusing on pediatrics, American Academy of Pediatrics recommends to obtain a laboratory confirmation of the presence of GABHS. In the decision to obtain a throat swab specimen, the clinician has to consider the age >3 years, clinical signs and symptoms of pharyngitis, the season and the community epidemiology, including contacts with GABHS infection or presence in the family of a person with a history of ARF or poststreptococcal glomerulonephritis. Children with signs or symptoms suggesting viral infection (coryza, conjunctivitis, hoarseness, cough, stomatitis or diarrhea) should not be tested [4].

Regarding the need to confirm a RADT negative result, Snow and Bisno suggest to perform a throat culture in children, while no other investigation is indicated in adults [14,15]. In reverse Gerber *et al *state that, if RADT is negative, a throat culture should be performed in both adults and children [13]. The need to confirm negative RADT results with a throat culture is advised also by American Academy of Pediatrics [4]. On the contrary, because of the high specificity, it is not necessary to confirm a positive RADT test [3].

It has been reported that RADTs are underused compared to the indications given in the American guidelines. A big retrospective USA study conducted by Linder *et al *including a total number of 4158 children with pharyngitis aged 3-17 years shows that physicians performed a GABHS test only in 63% of children with sore throat and prescribed antibiotics to 53% of children, exceeding the maximum expected prevalence of GABHS. There was a significant difference in antibiotic prescriptions between children who had a GABHS test performed and those who did not: GABHS testing is associated with a lower rate of antibiotic prescribing [26].

**Figure 4:**
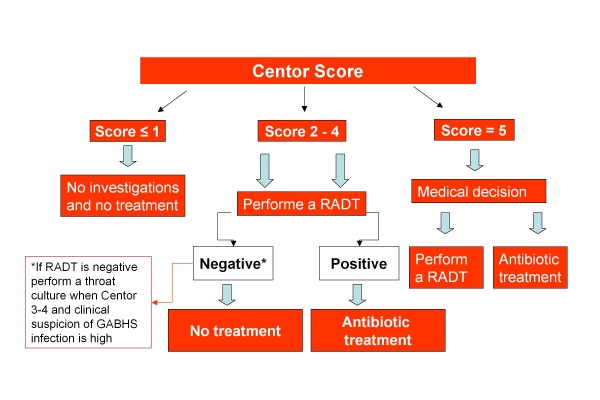
**Emilia Romagna regional guideline: flow-chart for management of pharyngitis **[18].

Considering Italy, the regional guideline of Emilia Romagna suggests to perform a RADT when Centor score ≥ 2. If RADT is positive then antibiotic treatment should be started; if RADT is negative and the clinical suspicion of GABHS pharyngitis is high, then a throat culture should be performed. When Centor score is 5, the physician should decide if starting the treatment directly or performing a microbiological test [18]. This flow chart is illustrated in figure 4[18].

## Treatment

As we explained before, antibiotic treatment is not routinely recommended, due to the prevalent viral etiology of pharyngitis. However, when antimicrobial treatment is indicated, it is important to choose a good therapeutic option.

All the authors and national guidelines agree in suggesting penicillin as first choice treatment, since GABHS remains universally susceptible to penicillin [3]. Although penicillin V is the drug of choice, ampicillin or amoxicillin equally are effective and, due to the good taste, represent a suitable option in children [4]. Moreover we have to remember that penicillin suspension is not commercially available in several countries including Italy, so that amoxicillin is usually prescribed.

Gerber *et al *state that prompt administration of penicillin therapy shortens the clinical course, decreases the incidence of suppurative sequelae, the risk of transmission and prevents ARF even when given up to 9 days after illness onset [13].

Therapeutic options with doses and duration recommended by American Academy of Pediatrics are illustrated in table 4[3].

**Table 4:** Therapeutic options for GABHS pharyngitis recommended by American Hearth Association and American Academy of Pediatrics AAP [13,4].

Drug	Dose	Duration
**Penicillins**		

**Penicillin V (oral)**	• Children <27 kg: 400 000 U (250 mg) 2 to 3 times daily ; • Children >27 kg, adolescents, and adults: 800 000 (500 mg) 2 to 3 times daily	10 days

**Amoxicillin (oral)**	50 mg/kg once daily (maximum 1 g)	10 days

**Benzathin Penicillin G (intramuscular)**	• Children <27 kg: 600 000 U (375 mg); • Children >27 kg, adolescents, and adults: 1 200 000 U (750 mg)	Once

**For individuals allergic to penicillin**		

Narrow-spectrum cephalosporin (cephalexin, cefadroxil) (oral)*	Variable	10 days

**Clindamycin (oral)**	20 mg/kg per day divided in 3 doses (maximum 1.8 g/d)	10 days

**Azithromycin (oral)**	12 mg/kg once daily (maximum 500 mg)	5 days

**Clarithromycin (oral)**	15 mg/kg per day divided BID (maximum 250 mg BID)	10 days

* Patients with immediate or type I hypersensitivity to penicillin should not be treated with a cephalosporin [4].

It is important to remember that macrolides are not indicated in the treatment of pharyngitis, due to the high rates of resistance to erythromycin among GABHS in USA and Europe [27]. Indication for the use of macrolides in pharyngitis is relegated to patients allergic to β-lactam antibiotics. The allergy should be proved by laboratory testing. If the patient's hypersensitivity to penicillin is not type I, cephalosporins should be considered a good therapeutic option [13].

The indication to use amoxicillin once daily, proposed by Gerber *et al *and widely employed in USA, is not universally accepted. Amoxicillin given once daily is not approved from Food and Drug Administration (FDA) and European Medicines Agency (EMEA) for primary profylaxis of ARF.

The standard duration of antibiotic therapy is 10 days. It has been proposed to shorten it to 3-6 days, so to improve the compliance [28]. A Cochrane review on 20 studies involving a total number of 13,102 cases of acute GABHS has been published in 2009. The authors compared short duration therapy (three to six days) of oral antibiotics (all types included) to standard duration treatment. They found that short duration treatment presented lower risk of early clinical treatment failure and no significant difference in early bacteriological treatment failure, or late clinical recurrence. Anyway, the overall risk of late bacteriological recurrence was worse in short duration treatment, although no significant differences were found when studies employing low dose azithromycin (10 mg/kg) were eliminated. Authors conclude that a short course (2 to 6 day) of oral antibiotics has an efficacy comparable to the standard duration therapy in treating children with acute GABHS pharyngitis [28]. Nevertheless the results of these review were largely criticized. Shad D.[29] underlines that at least one more eligible trial [30] and one meta-analysis [31] were not included. Besides, most of the trials included had methodological inaccuracy (i.e. randomization was not described or inappropriate in majority, only 3 of the 20 studies were blinded). Moreover ARF was considered as main outcome only in 3 of the 20 included studies with a total of 3 events recorded (insufficient power to make conclusions) [29]. Fagalas *et al *in a recent meta-analysis of Randomized Trials (8 RCTs, 1607 patients) found out that short-course treatment for GABHS pharyngotonsillitis is associated with inferior bacteriological eradication rates [31]. After an adequate therapy, follow-up cultures are not necessary unless symptoms recur [3].

Recurrent pharyngitis may represent a relapse or may result from new exposure [3]. In case of relapse cephalosporins have been proposed to be more effective than penicillin [32].

Some authors have suggested that cephalosporins could have an efficacy higher than penicillin on GABHS pharyngitis [33-35]. In a meta-analysis of 9 RCTs, involving 2113 adult patients with GABHS pharyngitis, Casey and Pichichero indicate that the likelihood of a bacteriologic and clinical cure of GABHS tonsillopharyngitis in adults is significantly higher after 10 days of therapy with an oral cephalosporin than with oral penicillin. They reported that the absolute difference in bacteriologic failure rates between cephalosporins and penicillin was 5.4% [33]. They also conducted a meta-analysis of RCT's of cephalosporin versus penicillin treatment of GABHS pharyngitis in children. It indicates that the likelihood of bacteriologic and clinical failure is significantly less if an oral cephalosporin is prescribed, compared with oral penicillin [34].

Anyway it must be remembered that no guidelines recommends cephalosporins as first choice drugs in the treatment of GABHS pharyngitis because of the higher cost compared to penicillin and the risk of selection of resistant strains. Their recommendation in guidelines is limited to patients with an hypersensibility to β-lactam non I type [36].

## Authors' opinion and conclusion

Correct diagnosis and treatment of GABHS pharyngitis are the key points to attain a judicious use of antibiotics, and to prevent suppurative and non suppurative sequelae. Thus, prudentially, we believe that paediatricians should perform at least one microbiological test (RADT or throat colture) in pharyngitis suspected for GABHS etiology, in order to make the correct diagnosis. Most RADTs can provide results in few minutes and their sensitivity is generally high [4]. Practically, we suggest that a negative RADT should be confirmed by a throat culture only if clinical suspicion of GABHS pharyngitis is high. Pharyngitis of proved bacterial etiology should receive an antibiotic treatment [4,13-15]. Penicillin V is the first choice drug, but an oral suspension is not available in Italy. Amoxicillin is equally effective and demonstrates higher palatability, so that it can be used as first line therapy [4,15]. Macrolides are not indicated in the treatment of GABHS pharyngitis except for patients with a proved allergy to penicillin (laboratory confirmation should be required) [4,15]. For this group of patients Cephalosporins represent a good alternative (left out cases of type I hypersensitivity to penicillin) [4,15]. The inappropriate use of macrolides for treatment of GABHS pharyngitis has been the main cause of resistant strains diffusion in Western countries [27]. It is important to underline that treatment duration should be 10 days [4,15]. To improve the patient's compliance the physician should explain the importance of the complete treatment (10 days) to eradicate the bacterium even if clinical improvement occurs in the first 4-5 day of treatment.

To date no Italian guideline is available, but we believe that it should be fundamental to establish a rational and uniform approach to the management of acute GABHS pharyngitis in all the country.

## Competing interests

The authors declare that they have no competing interests.

## Authors' contributions

MR, EC, FB performed the literature search, data interpretation and wrote the manuscript. LG and MdM performed the data interpretation and the final revision of the manuscript. All authors read and approved the final manuscript.
